# Wheat dwarfing influences selection of the rhizosphere microbiome

**DOI:** 10.1038/s41598-020-58402-y

**Published:** 2020-01-29

**Authors:** Vanessa N. Kavamura, Rebekah J. Robinson, David Hughes, Ian Clark, Maike Rossmann, Itamar Soares de Melo, Penny R. Hirsch, Rodrigo Mendes, Tim H. Mauchline

**Affiliations:** 1 Sustainable Agriculture Sciences, Rothamsted Research, Harpenden, Hertfordshire, United Kingdom; 2 Plant Pathology Laboratory, Royal Horticultural Society, RHS Garden Wisley, Woking, Surrey, GU23 6QB United Kingdom; 3 Computational and Analytical Sciences, Rothamsted Research, Harpenden, Hertfordshire, United Kingdom; 4 Laboratory of Environmental Microbiology, Embrapa Environment, Jaguariúna-SP, Brazil

**Keywords:** Soil microbiology, Microbiome

## Abstract

The development of dwarf wheat cultivars combined with high levels of agrochemical inputs during the green revolution resulted in high yielding cropping systems. However, changes in wheat cultivars were made without considering impacts on plant and soil microbe interactions. We studied the effect of these changes on root traits and on the assembly of rhizosphere bacterial communities by comparing eight wheat cultivars ranging from tall to semi-dwarf plants grown under field conditions. Wheat breeding influenced root diameter and specific root length (SRL). Rhizosphere bacterial communities from tall cultivars were distinct from those associated with semi-dwarf cultivars, with higher differential abundance of Actinobacteria, Bacteroidetes and Proteobacteria in tall cultivars, compared with a higher differential abundance of Verrucomicrobia, Planctomycetes and Acidobacteria in semi-dwarf cultivars. Predicted microbial functions were also impacted and network analysis revealed a greater level of connectedness between microbial communities in the tall cultivars relative to semi-dwarf cultivars. Taken together, results suggest that the development of semi-dwarf plants might have affected the ability of plants to recruit and sustain a complex bacterial community network in the rhizosphere.

## Introduction

Wheat domestication originated in the Near East and the crop has undergone a series of crosses and modifications leading to the hexaploid bread species, *Triticum aestivum* L^1^. Wheat is a key crop for global food security, providing 20% of dietary requirements for calories and protein^2^. To achieve food demand of an increasing global population, projections forecast a need to increase wheat production by 11% by 2026 with just 1.8% increase in cultivation areas^3^. During the Green Revolution, Reduced height (*Rht*) dwarfing genes were introduced in modern wheat cultivars^4^, resulting in high-yielding wheat plants, which, when combined with agrochemical management and optimal conditions, increased yields, without productivity losses caused by lodging^5^. Plant height is a complex trait controlled by several Quantitative Trait Loci (QTLs) and more than 20 *Rht* genes have been described, however, most of them have low potential for successful breeding programs^6^. Most breeding programs take into consideration the improvement of above ground parts of plants, with little attention to below ground parts due to the inherent challenges of root analysis coupled with the influence of soil type on root traits^7,8^. As such, the effect of domestication and modern breeding on belowground traits in wheat largely remains unclear. However, Bai *et al*.^9^ assessed a diverse set of wheat germplasm [199 double-haploid progeny derived from a cross between Avalon and Cadenza (*Triticum aestivum* L.)] and found that plant height was positively correlated to some evaluated root traits such as: total root length, seminal laterals length, seminal axes length, seminal laterals surface area, seminal laterals volume and root dry weight. In addition, Figueroa-Bustos *et al*.^10^ reported a positive correlation between plant height and specific root length (SRL). The selection of desirable root traits through “root breeding”, would enable plants to explore soil more efficiently, thus improving water and nutrient acquisition^11^. It has also become clear that future breeding programs should consider plant-microbe interactions^12^ as plants are complex holobiont organisms influenced both positively and negatively by their microbial communities^13^.

It is now known that the wheat microbiome can be influenced by host genotype^14,15^, fertilization regime^16^, land management and seed load^17^, irrigation^18^, seed germination and sterilization^19^, tissue type and growth stage^20^, plant organ, host age and management strategy^21^. However, there are few studies correlating root traits with microbiome structure. Pérez-Jaramillo *et al*.^22^ found that 11.4% of bacterial community composition was explained by root traits, e.g. SRL, in common bean cultivars. Saleem *et al*.^23^, studying the impact of root system architecture on rhizosphere and root microbiome, found that fine roots harboured higher microbial richness than secondary and primary roots of nicotiana cultivars.

The selective breeding of wheat cultivars led to structural changes in wheat^24,25^, which could also have affected associated microbial communities. Similar to the gut microbiome, which is considered to play an important role in host health^26^, the microbiome of plants helps them tolerate biotic and abiotic stresses^27^. The hypothesis that plant breeding has influenced microbial communities of wheat was previously tested by Germida and Siciliano^28^, who addressed the diversity of culturable bacteria from ancient and modern wheat cultivars. Due to technological limitations, a deeper total community analysis was not possible in their study, however, with the development of high throughput next generation sequencing technologies the study of complex microbial communities can now be achieved in unprecedented detail.

We hypothesize that wheat breeding has affected root morphological traits, impacting bacterial community structure, diversity and 16S rRNA gene-predicted functions in the wheat rhizosphere. We tested this hypothesis by comparing the rhizosphere bacterial communities from a range of tall and semi-dwarf wheat plants grown under field conditions.

## Material and Methods

### Rhizosphere sampling of wheat accessions

A “heritage wheat” experiment at Rothamsted Research consisted of hexaploid wheat varieties planted in triplicate in 1 m^2^ plots randomly designed with approximately 350 seeds per plot. Nitrogen was applied as ammonium nitrate at 210 kg/ha with other inputs according to standard agronomic practice. Three plants at flowering stage were harvested from each plot in July (2014), and rhizosphere soil was collected by gently discarding loose soil and vigorously shaking the roots inside a polythene bag to release tightly attached soil, which was considered as rhizosphere^16^. Rhizosphere soil was homogenized, and a 5 g subsample was taken and stored at −80 °C prior to soil DNA extraction. Experimental design consisted of 8 cultivars × 3 replicates, resulting in 24 samples. Varieties range from Chidham White Chaff, a long straw variety from 1790 to the modern variety Crusoe from 2012. These varieties were grouped into two categories: “Tall” representing cultivars which were obtained before the Green Revolution and “Semi-dwarf” representing cultivars of which functional Reduced height *(Rht)* dwarfing genes have been incorporated by selective breeding (Table 1) (Supplementary Fig. S1).

**Table 1:** Cultivars chosen for the current study and some characteristics, such as year of release, pedigree and height.

Cultivar	Year	Pedigree	Height^a^
Chidham White Chaff	1790	Not recorded	Tall
Red Lammas	1850	Not recorded	Tall
Victor	1908	(Squarehead*Red King)*Talavera	Tall
Avalon	1980	TJB 30/148* TL 365a/34/5	Semi-dwarf
Hereward	1989	Norman’sib’*Disponent	Semi-dwarf
Malacca	1997	Riband*(Rendezvous)*Apostle	Semi-dwarf
Gallant	2009	(Malacca*Charger)*Xi-19	Semi-dwarf
Crusoe	2012	Cordiale*Gulliver	Semi-dwarf

^a^Height was based on field data collected by Shewry *et al*.^29^ and was categorised as described by Pask *et al*.^30^.

### Root morphological traits

Wheat seeds were surface sterilised following the protocol by Robinson *et al*.^31^ and pre-germinated in Petri dishes with filter paper soaked in autoclaved distilled water. Analysis of root morphology was performed according to Bai *et al*.^9^. Full details are given in Supplementary Methods. Specific root length (SRL) and statistical analyses were calculated in R (version 3.5.0) as described by Pérez-Jaramillo *et al*.^22^. Normality and homogeneity of variances were checked using Shapiro–Wilk test and Levene’s test, respectively, and One-way ANOVA and post-hoc test (Tukey HSD) were used to assess differences.

### Soil DNA extraction and quantification

For each sample, DNA was extracted from 0.25 g of rhizosphere soil using the MoBio PowerSoil™ DNA Isolation Kit (Carlsbad, CA, USA). Extractions were performed according to the manufacturer’s instructions but with the use of the MP Biomedicals FastPrep-24 machine twice for 30 s at 5.5 m.s^−1^. DNA purity and concentration were determined by NanoDrop spectrophotometry (Thermo Scientific, Wilmington, DE, USA) as well as a Qubit 2.0 Fluorimeter using ds DNA HS assay kit (Thermo Fisher).

### Illumina bacterial 16S rRNA gene sequencing

Briefly, PCR amplicon libraries targeting the 16S rRNA encoding gene present in metagenomic DNA were produced using a barcoded primer set adapted for the Illumina HiSeq. 2000 and MiSeq^32^. DNA sequence data was generated using Illumina paired-end sequencing at the Environmental Sample Preparation and Sequencing Facility (ESPSF) at Argonne National Laboratory. Full details are given in Supplementary Methods.

### Metataxonomic sequence analysis pipeline

16S rRNA gene sequences were analysed using the pipeline proposed by the Brazilian Microbiome Project (BMP) available at http://brmicrobiome.org ^33^ with some modifications. Details are described in Supplementary Methods. It uses Quantitative Insights Into Microbial Ecology (QIIME) (version 1.8.0)^34^ and USEARCH 9.0^35^. Operational taxonomic units (OTUs) were defined to 97% sequence identity against SILVA 128 database^36^. The generated OTU table was filtered using QIIME scripts to extract Bacterial domain and remove chloroplasts as suggested by Pérez-Jaramillo *et al*.^22^. Differences in bacterial community structure were investigated by Permutational Analysis of Variance (PERMANOVA)^37^ in Paleontological Statistics Software Package for Education and Data Analysis (PAST)^38^. PCoA plots were obtained using the same software. The number of observed OTUs and diversity based on Shannon index were calculated in QIIME. Statistical analyses of alpha diversity indexes were performed in R as described by Pérez-Jaramillo *et al*.^22^. Briefly, normality and homogeneity of variances were checked using Shapiro–Wilk test and Levene’s test, respectively, and One-way ANOVA and post-hoc test (Tukey HSD) were used to assess differences.

### Analysis of differentially abundant OTUs

The online tool for comprehensive statistical, visual and meta-analysis of microbiome data called Microbiome Analyst^39^ was used for detecting OTUs which were differentially abundant between tall and semi-dwarf cultivars, using DESeq. 2, which has high sensitivity for small datasets (<20 samples per group). Besides, False Discovery Rate (FDR) values are relatively low for data sets with similar sizes when compared to other tools such as metagenomeSeq^40^. Further details are supplied in Supplementary Methods.

### Network analysis

Network analyses were performed using the Molecular Ecological Network Analyses (MENA) pipeline^41^ available at http://ieg4.rccc.ou.edu/mena/. Network topological properties were calculated and evaluated^41^. Phylogenetic molecular ecological networks (pMENs) were visualised using Cytoscape (v.3.4.0)^42^. Full details are provided in Supplementary Methods. Additionally, co-occurrence analyses were performed using python module SparCC^43^. For this, the filtered OTU table previously obtained (with at least 0.01% of total abundance) was used. For each network, P-values were obtained by 100 permutations of random selections of the data table. Statistically significant (p < 0.01) SparCC correlations with a magnitude of > 0.7 or < −0.7 were included into the network analyses. Networks were visualized with Cytoscape (v.3.4.0)^42^.

### Functional prediction from 16S rRNA gene data

In addition to the taxonomic analysis, 16S rRNA gene libraries were used to predict metagenome function using the PICRUSt software^44^ within the GALAXY server provided by the Langille Lab (v1.1.1) (http://galaxy.morganlangille.com/). Details on PICRUSt analyses are given in Supplementary Methods. The table with 16S rRNA gene-predicted functions was exported into Statistical Analysis of Metagenomic Profiles (STAMP) software (version 2.1.3)^45^ to test for statistical differences with ANOVA. Extended error plots were obtained, and they show the proportion of sequences (%) that were significantly different using a post-hoc test (Tukey-Kramer), Eta-squared, to measure effect size and Benjamini-Hochberg-FDR as a multiple test correction (p < 0.05). Relevant KEGG orthologs (KOs) were selected for further analyses.

## Results and Discussion

### Effect of wheat breeding on root morphological traits

Breeding process appears to have altered some root traits, especially root diameter and specific root length (SRL). Tall plants (old cultivars), when compared to semi-dwarf plants (recent cultivars), showed statistically higher mean SRL (tall = 113.63 m.g^−1^ and semi-dwarf = 91.62 m.g^−1^) (One-Way ANOVA, p < 0.05). The opposite general trend is observed for root diameter (tall = 0.44 mm and semi-dwarf = 0.49 mm) (One-Way ANOVA, p < 0.05) (Supplementary Fig. S2). Tall cultivars tend to have longer thinner roots, whereas shorter thicker roots are observed for semi-dwarf cultivars. Pérez-Jaramillo *et al*.^22^ also observed higher SRL values for wild common bean accessions, which could be useful for enhanced water and nutrient uptake in stressed soils, than the lower SRL of short varieties, suggesting semi-dwarf cultivars could be more dependent on fertile soils. Indeed, modern Italian bread wheat cultivars increased their N demand over time when compared to old tall cultivars^46^. Plant height reduction was achieved during the 20th century wheat breeding^47^, though little attention was given to assessing the effects of these programmes on wheat root traits. In an attempt to study whether *Rht* genes control both shoot height and seedling root growth, Bai *et al*.^9^ evaluated QTLs for plant height, root and seed traits from different wheat lines and identified some coincident QTLs for roots and height, concluding that the introduction of some known *Rht* dwarfing genes reduced both plant height and root proliferation. Breeding for higher yields in Australian wheat cultivars led to a reduced root length density (RLD) and total root length and increased efficiency of N uptake^48^. For other crops, such as barley, common bean, rice and maize, it has also been shown that both domestication and breeding caused changes in root architectural traits, leading to a differential spatial arrangement of roots^49^.

### Effect of breeding process on bacterial structure, richness, diversity and differential recruitment of bacterial taxa

**Figure 1:**
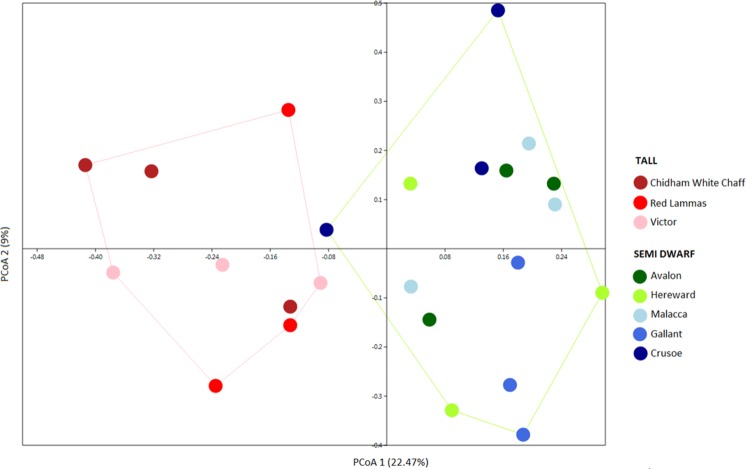
PCoA based on Bray-Curtis similarity distance matrix showing the structure of wheat rhizosphere bacterial communities associated with tall (dark red, red and pink) and semi-dwarf cultivars (green and blue).

Bacterial communities from the wheat rhizosphere were mainly represented by Proteobacteria (36–41.7%), followed by Actinobacteria (12.1–16.1%), Bacteroidetes (11.1–16%) and Acidobacteria (8.7–13%) (Supplementary Fig. S3). The PCoA plot shows rhizosphere bacterial communities from tall cultivars differ from those associated with semi-dwarf cultivars (PERMANOVA, F = 0.827, p = 0.0001), with the first PCoA axis corresponding to 22.47% of the variation (Fig. 1). In general, tall plants when compared to semi-dwarf plants showed lower diversity (based on Shannon index) (tall = 9.068 and semi-dwarf = 9.423) and lower number of observed OTUs (tall = 1642.289 and semi-dwarf = 1810.667) (Supplementary Fig. S4).

**Figure 2:**
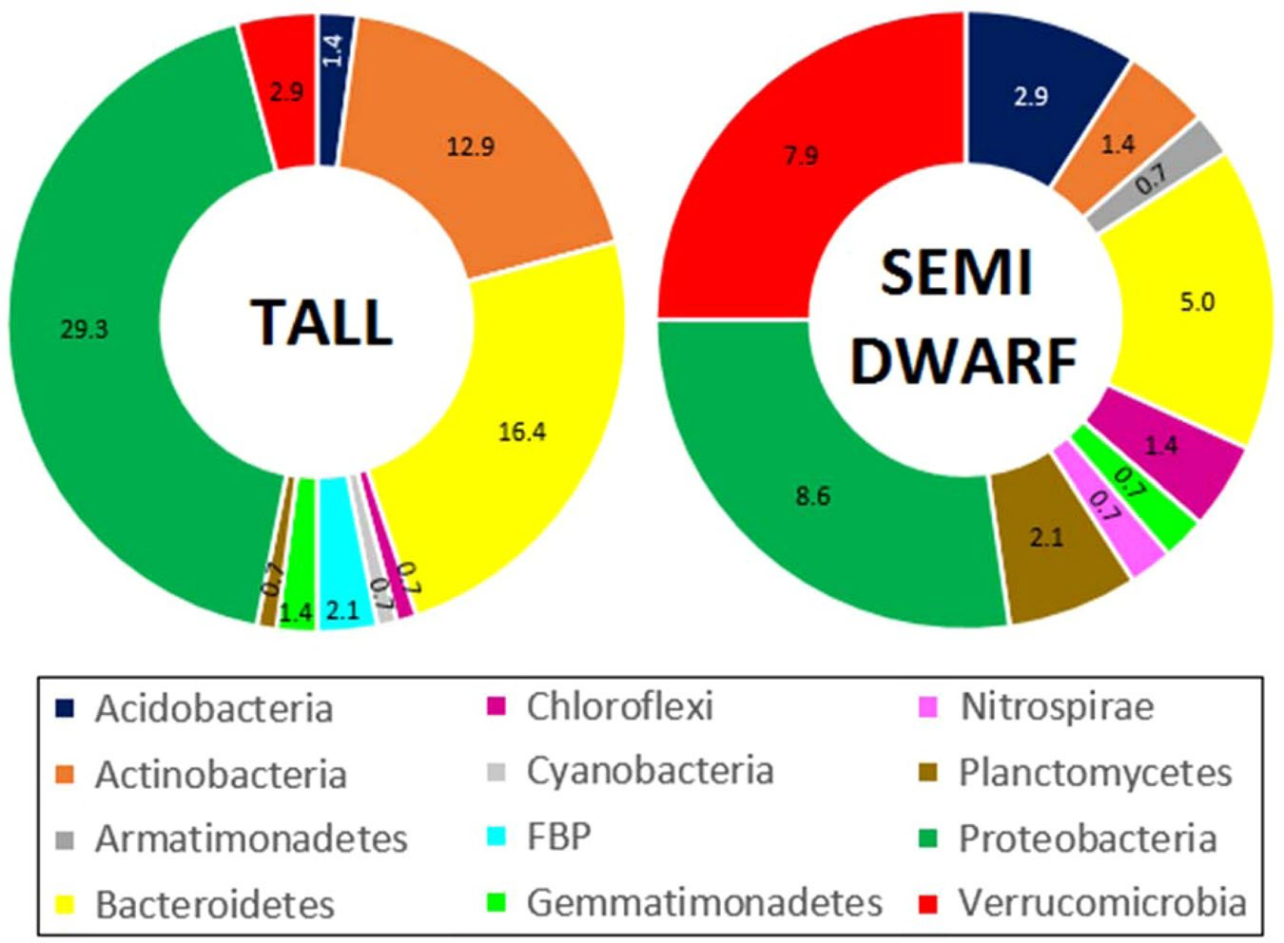
Doughnut charts showing the values (%) of differentially abundant OTUs grouped at phylum level, from a total of 140 detected among tall and semi-dwarf cultivars.

From a total of 140 significantly differentially abundant OTUs detected with DESeq. 2, 29.3% of OTUs belonging to the phylum Proteobacteria were significantly more abundant in the rhizosphere of tall cultivars, as opposed to 8.6% associated to semi-dwarf cultivars (Fig. 2).

Tall cultivars also showed a higher percentage of differentially abundant OTUs belonging to the phyla Bacteroidetes (16.4%), Actinobacteria (12.9%) and Gemmatimonadetes (1.4%). On the other hand, semi-dwarf cultivars showed a larger proportion of OTUs belonging to the phyla Verrucomicrobia (7.9%), Acidobacteria (2.9%), Planctomycetes (2.1%) and Chloroflexi (1.4%). Additionally, OTUs differentially abundant and assigned to Armatimonadetes and Nitrospirae were only observed in semi-dwarf cultivars (0.7% in both cases) and significantly more OTUs assigned to Cyanobacteria and FBP were only detected in association with tall cultivars (0.7% and 2.1%, respectively).

The rhizospheres of semi-dwarf cultivars are enriched in members belonging to the phyla Acidobacteria and Verrucomicrobia, which are typically more associated with bulk soil rather than the wheat rhizosphere^16^. A closer analysis at genus level, indicates that tall cultivars are enriched with OTUs assigned to genera normally associated with plant growth promotion (PGP), especially members of the phylum Proteobacteria (Table 2) such as *Brevundimonas* (indole-3-acetic acid (IAA) production and P solubilisation)^50^; *Devosia* (N_2_ fixation)^51^; *Rhizomicrobium* (reduction of nitrate to nitrite)^52^; *Sphingomonas* (IAA and gibberellin production)^53^; *Sphingopyxis* (IAA production and N_2_ fixation)^54^; *Massilia* (IAA, siderophore and proteolytic enzyme production)^55^; *Nitrosospira* (ammonia-oxidizing bacterium (AOB))^56^ and *Bradyrhizobium, Rhizobium, Methylobacterium, Variovorax, Klebsiella* and *Pseudomonas* which typically possess genes contributing to plant-beneficial functions^57^. Although some differentially recruited OTUs within tall rhizospheres have been assigned to genera commonly related to plant growth promotion, these abilities are normally strain-specific, thus caution must be taken when analysing the results. Further work should evaluate these differences based on metagenomic datasets as well as functional characterisation of microbial isolates.

**Table 2:** Proteobacteria genera assigned to OTUs which were found to be enriched in the rhizosphere of tall and semi-dwarf cultivars.

Height	Class	Genus
Tall	Alphaproteobacteria	*Bradyrhizobium, Brevundimonas, Devosia*,
		*Methylobacterium, Rhizobium, Rhizomicrobium*,
		*Skermanella, Sphingomonas, Sphingopyxis*,
		*Variibacter*, uncultured Caulobacteraceae,
		unclassified Aurantimonadaceae, unclassified Rhizobiaceae,
		unclassified Rickettsiales and unclassified Sphingomonadaceae
	Betaproteobacteria	*Massilia, Nitrosospira, Verticia, Variovorax*,
		uncultured order B1-7BS, unclassified Oxalobacteraceae and
		unclassified Comamonadaceae
	Deltaproteobacteria	*Bacteriovorax*
	Gammaproteobacteria	*Dyella, Klebsiella, Lysobacter*,
		*Pseudomonas, Rhodanobacter* and *Stenotrophomonas*
Semi-dwarf	Betaproteobacteria	Uncultured Alcaligenaceae and uncultured Nitrosomonadaceae
	Deltaproteobacteria	*Haliangium*, Phaselicystidaceae and uncultured Myxococcales
	Gammaproteobacteria	Polycyclovorans and uncultured Xanthomonadales

The major metabolic consequence of plant dwarfing is a reduction in response to the plant phytohormone gibberellin^58^ and an increase of active endogenous gibberellin levels when compared to wild-type plants not carrying *Rht* dwarfing genes^59,60^. The correlation that the rhizosphere of short plants with decreased gibberellin sensitivity is associated with an increased colonisation of bacteria normally associated with bulk soil and a decrease of microbial phyla associated with plant growth promotion, suggests that gibberellin sensitivity influences the composition of the root microbiome. It is known that some microbes, such as PGP members of Rhizobiaceae family^61^ as well as some *Sphingomonas* spp^53^. produce this hormone, and we find these bacteria to be differentially more abundant in tall cultivar rhizospheres. It could follow that plant gibberellin insensitivity leads to a reduction in plant-microbe communication in the root environment and could reduce selection of gibberellin-producing microbes in the root zone. Apart from controlling the growth and development of plants, gibberellins also play a role in plant signalling^60^, affecting defence response mechanisms dependent on jasmonic acid (JA) or salicylic acid (SA)^62^, rhizobial infection of legumes^63^ mediated via DELLA proteins and can suppress arbuscular colonisation and development of mycorrhizal symbiosis^64^. Work should be conducted to determine if the root microbiome of these crop accessions diverges further when tall and shorts cultivars are cultured under low nutrient conditions. This would support the hypothesis that the tall cultivars are more capable of recruiting beneficial microbes, and when under stress they do this more readily than short cultivars. Furthermore, microbes have been shown to be capable of producing a range of phytohormones^65^. It will be interesting to determine if the plant breeding process has influenced the plant hormone status for other classes of phytohormones including abscisic acid, auxins, brassinosteroids, ethylene, jasmonates, salicyclic acid and strigolactones, as there is a strong interaction among them^66^ and whether their levels influence microbiome composition.

In addition to plant hormonal levels, the observed differences in microbiome structure between short and tall cultivars could be attributed to root exudation profiles^23^. Iannucci *et al*.^67^ showed that even though soil type dramatically changed the composition of root exudates, domestication and breeding also had major effects on root exudates in the rhizosphere of ten tetraploid wheat genotypes. The sensing and metabolism of root metabolites are likely important factors in determining microbiome assembly^68^.

### Effect of wheat breeding on key rhizosphere taxa and network complexity

**Figure 3:**
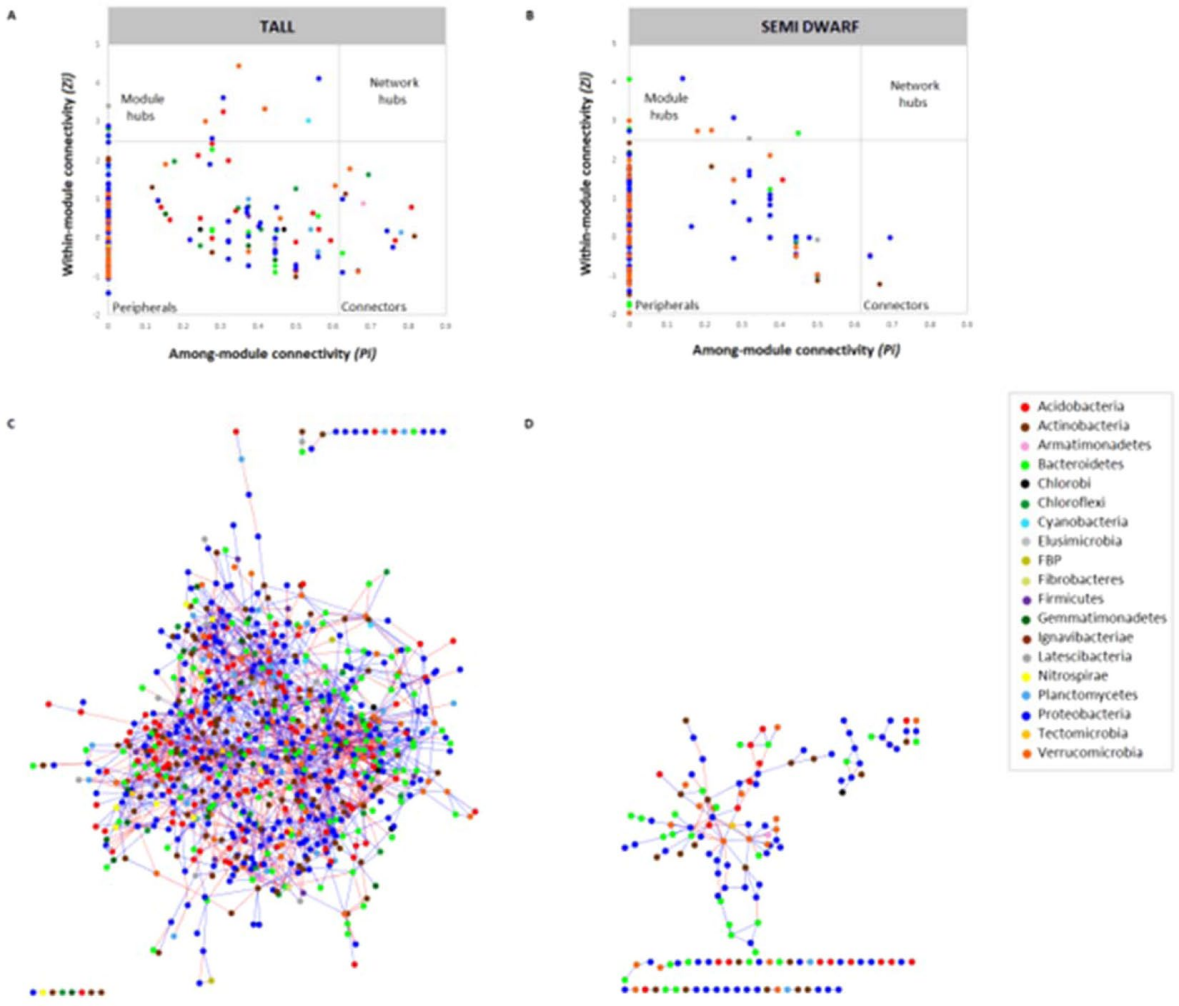
Classification of nodes (OTUs) to identify putative keystone OTUs within rhizosphere networks from tall (A) and semi-dwarf (B) cultivars. Node topologies are placed into four categories: **module hubs** (*Zi* > 2.5) are highly connected nodes within modules; **connectors** (*Pi* > 0.62) are nodes responsible for connecting modules; **network hubs** (*Zi* > 2.5 and *Pi* > 0.62) are highly connected nodes within the entire network and **peripherals** (*Zi* < 2.5 and *Pi* < 0.62) are nodes connected in modules with few outside connections. Bacterial co-occurrence networks from tall (C) and semi-dwarf (D) cultivars, calculated with sparCC, with correlations with a magnitude of > 0.7 or < −0.7. Each node (dot) represents an OTU and each colour indicates one respective phylum. The edges are represented by lines, with positive co-occurrence patterns between two nodes shown in blue and negative co-occurrence patterns shown in red.

We found that for both tall and semi-dwarf networks, zero network hubs were identified and that most nodes were categorised as peripherals (Fig. 3A,B). However, fifteen nodes were classified as module hubs in tall cultivars and they represented a variety of taxonomic groups: five belonged to Proteobacteria (genera *Pseudomonas, Luteibacter, Sorangium* and orders Myxococcales and Xanthomonadales), three to Verrucomicrobia, two to Acidobacteria and one each from Bacteroidetes, Chloroflexi, Cyanobacteria, Gemmatimonadetes and Latescibacteria. Ten module hubs were identified in semi-dwarf cultivars and three belonged to Proteobacteria (one from ß-Proteobacteria, one from the family Rhodospirillaceae and one from the family Nitrosomonadaceae), three from Bacteroidetes (genera *Parafilimonas* and *Fluviicola* and family Saprospiraceae), three from Verrucomicrobia and one from Latescibacteria. Connectors were also detected in both cases, however, semi-dwarf cultivars displayed only four connectors, identified as belonging to genera *Asticcacaulis* and *Pseudohongiella*, both from phylum Proteobacteria, genus *Luedemannella* (Actinobacteria) and one Acidobacteria genus (RB41). On the other hand, tall cultivars showed fifteen connectors: two Acidobacteria (classes Subgroup 22 and Holophagae), one Actinobacteria (class MB-A2-108), one Armatimonadetes (family Fimbriimonadaceae), one Bacteroidetes (genus *Pedobacter*), one Chlorobi (order Ignavibacteriales), one Chloroflexi (class S085), one Planctomycetes (family Tepidisphaeraceae), two Verrucomicrobia (one identified as the genus *Chthoniobacter* and other belonging to the family Verrucomicrobiaceae) and five Proteobacteria (two belonging to the genus *Haliangium*, one betaproteobacterium from the family Rhodocyclaceae, one deltaproteobacterium from the family Sandaracinaceae and one α-Proteobacterium from the order Rhodospirillales (family DA111)). Members of the phylum Proteobacteria were found to be the most prominent putative keystone taxa, accounting for more than 30% of all module hubs and connectors in both tall and semi-dwarf cultivar networks. Members of Bacteroidetes and Verrucomicrobia were the second most prominent keystone taxa for semi-dwarf cultivars, each accounting for 21.43% of module hubs. On the other hand, members of Bacteroidetes accounted for less than 7% of module hubs and connectors and Verrucomicrobia members accounted for 16.67% in tall cultivars. Module hub and connector OTUs have been proposed as putative keystone taxa and ecological generalists, critical for maintaining community structure and function; peripherals on the other hand being considered isolated specialists^41,69–74^. As such, Proteobacteria seem to be critical in maintaining the structure and functionality of bacterial communities in wheat rhizosphere for both tall and semi dwarf cultivars and these results are in accordance with data provided by Oberholster *et al*.^71^, who also found Proteobacteria to be important keystone taxa for sorghum and sunflower rhizospheres.

Phylogenetic molecular ecological networks (pMENs) were obtained for both tall and semi-dwarf cultivars and the overall structure of each pMEN is presented in Supplementary Table S1. Topological properties indicated that R^2^ values for both networks (0.907 for tall cultivars and 0.899 for semi-dwarf cultivars) followed a power-law model, indicating that only a few nodes have many connections with other nodes^41^. Average path distance (GD) obtained for tall cultivars was lower than semi-dwarf cultivars, indicating that nodes in the network from tall cultivars are closer to each other. The average degree (avgK) obtained for tall cultivars indicated a more complex network structure, as a higher value was obtained. This higher complexity was also highlighted by networks calculated with sparCC represented in Fig. 3C,D. High values of modularity for both networks (tall = 0.749 and semi-dwarf = 0.890) indicate that they can be separated into modules using fast greedy modularity optimisation. Modularity values range from 0 to 1 and they measure the degree to which the network was organised into clearly delimited modules^74^ and modular structure of complex networks display critical roles in their functionality^75^. The number of modules obtained for tall cultivars was ninety-four, whereas semi-dwarf cultivars resulted in one-hundred and fifteen modules. A module is defined as a group of OTUs which are highly connected, however these are poorly connected with OTUs outside a given module^41^. We found that modules comprised of 4 nodes or more numbered 38 for dwarf varieties compared to 21 for tall varieties, indicating that the OTUs are better connected within the tall cultivar network (Supplementary Fig. S5**)**.

The number of edges obtained in the pMEN from bacterial communities from tall cultivars was higher than those from semi-dwarf cultivars and this was also highlighted by the connectedness index which was 0.498 as opposed to 0.293 for semi-dwarf cultivars. This index ranges from 0 to 1, with values close to 1 indicating a highly-connected graph^41^. The ratio of positive to negative co-occurrence patterns for tall cultivars was 3.094, whereas the ratio of positive to negative interactions for semi-dwarf cultivars was 5.331, indicating that bacterial communities from semi-dwarf cultivars displayed more positive co-occurrence relationships than tall cultivars. The composition and number of nodes per modules varied and the composition of modules is shown in Supplementary Fig. S6. Similarly to nodes identified as keystone taxa, the majority of nodes were classified as Proteobacteria, followed by Acidobacteria, Bacteroidetes, Actinobacteria and Verrucomicrobia. For tall cultivars, the biggest module (#6) comprised of 70 nodes, with more than 32% of nodes belonging to Proteobacteria. As for semi-dwarf cultivars, the largest module (#1) was composed of 71 nodes, with more than 35% belonging to Proteobacteria.

In the present study, tall cultivars showed bacterial lower diversity than semi-dwarf cultivars as well as a more complex bacterial network structure in the rhizosphere. Shi *et al*.^69^ observed that microbial diversity decreased as network size and connectivity increased, which resulted in an increased community organisation. It has also been shown that more complex networks are able to cope with environmental changes^76^, increase crop productivity^73^ or suppress soil borne pathogen infection on plants^77^. Indeed, semi-dwarf wheat cultivars carrying *Rht* alleles were previously shown to be more susceptible than wild cultivars to initial infection by some pathogens^78,79^. This might be linked to the severity of DELLA effect on plant stature, however, differences in differential abundance of specific bacterial keystone taxa might point out some microbial influence on disease susceptibility. For instance, in the network from tall cultivars, OTUs 1135 and 427 in modules 2 and 3, respectively, were classified as belonging to the genus *Haliangium*, whose species have been described as putative biocontrol agents^80^ and another three-keystone species, from the same order, Myxococcales, were also observed in tall cultivars. Myxobacteria include Gram-negative gliding bacteria with predatory features, mostly due to the direct cell to cell contact, production of hydrolytic enzymes and secondary metabolites with antibiotic activity^81^. The third module in tall cultivars show one module hub (OTU 575 – classified as belonging to the genus *Chryseobacterium*) which has previously been described in the rhizosphere of wheat [14, 82;], and these bacteria have been shown to display plant growth-promotion^82^ and are enriched in the presence of 2,4-DAPG-producing species, such as *Pseudomonas fluorescens*^83^. Interestingly, the other module hub identified in the same module as *Chryseobacterium* was OTU 171 (assigned to the genus *Pseudomonas*) and they positively co-occur, meaning that their abundance changed along the same trend but not necessarily meaning they directly interact with each other^74^. OTU 280, assigned to the genus *Pedobacter* and OTU 563 (*Luteibacter*) have also been identified as keystone taxa in tall cultivars. They have been found to inhibit the growth of root pathogens, such as the fungus *Rhizoctonia solani*^84,85^. When comparing both networks, only two OTUs (601 and 118) assigned to the genus RB41 (Blastocatellaceae family from the phylum Acidobacteria) were commonly found and were identified as keystone species. The breeding process seems to have reduced the amount of keystone taxa known for their potential to antagonise pathogens, possibly affecting the degree of resistance of semi-dwarf cultivars to specific diseases. Future work should investigate whether tall and semi-dwarf cultivars select different beneficial bacteria with PGP abilities, as well as antagonistic activities against economically important soil-borne pathogens.

### Effect of wheat breeding on 16S rRNA gene-predicted functions

**Figure 4:**
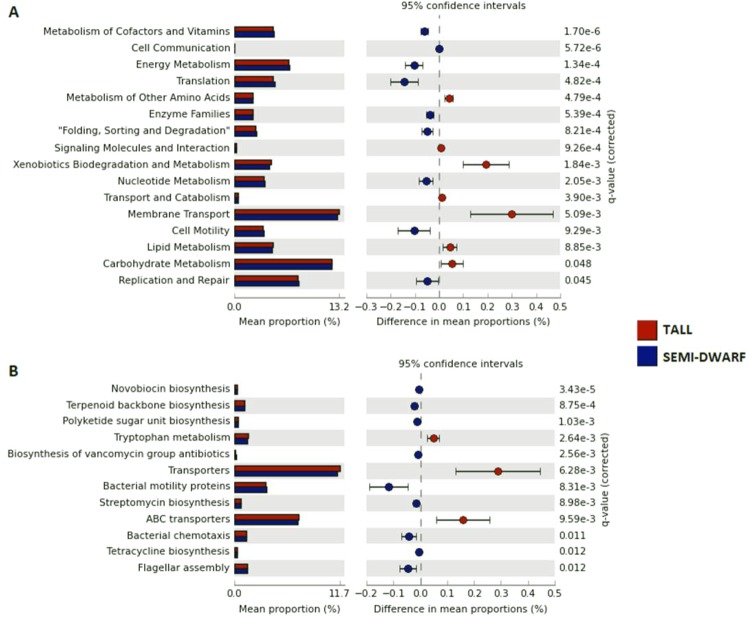
Statistical comparison using Welch’s t-test between the 16S rRNA-predicted functions of rhizosphere samples associated to tall and semi dwarf cultivars using Benjamini-Hochberg FRD correction (p < 0.05), showing the significant pathways (**A**) and specific selected KOs (**B**).

NSTI values obtained for 16S rRNA gene-predicted functions on rhizosphere soil samples are on average 0.1679 ± 0.013 for all samples and this value is in accordance to what is observed for soils^44^ and is lower when compared to other rhizosphere soils^86^. Rhizosphere samples collected from tall cultivars, on average have a NSTI value of 0.1534 ± 0.007 which is lower than NSTI value obtained from the rhizosphere of semi-dwarf cultivars (0.1766 ± 0.006). Functional prediction resulted in two hundred and twenty-one KEGG orthologs (KOs), of which one hundred and forty-one were differentially abundant (p < 0.05). Of these, 45.39% were significantly more abundant in tall cultivars and the remaining 54.61% were significantly more abundant in semi-dwarf cultivars. Seven 16S rRNA gene-predicted pathways were significantly enriched in rhizosphere bacterial communities of tall cultivars, as opposed to nine pathways enriched in the rhizosphere of semi-dwarf cultivars (Fig. 4A). Tall cultivars were predicted to be enriched in functions related to membrane transport, such as ABC transporters (Fig. 4B). Mahoney *et al*.^14^ suggested that differences in 16S rRNA gene-predicted functions observed between wheat cultivars could be driven by differential root exudate chemistry. The breeding process may have affected the quality and quantity of root exudates, impacting predicted functions in the rhizosphere. The presence of bespoke solute binding protein-dependent ABC transporters may be required for the use of root exudates by bacteria, aiding their colonisation of this niche^87^. When analysing specific KOs, tryptophan metabolism was also predicted to be enriched in bacterial communities from the rhizosphere of tall cultivars (Fig. 4B) and this suggests higher production of indole-3-acetic acid (IAA) hormone, as tryptophan is the main precursor for IAA biosynthesis, acting as a signalling molecule between plants and microbes as well as for plant growth promotion^88^. It could also mean that tall wheat plants are secreting more tryptophan upon colonization by specific bacterial groups^89^. On the other hand, bacterial communities from semi-dwarf cultivars were predicted to be enriched in predicted functions related to cell motility, such as bacterial motility proteins, bacterial chemotaxis and flagellar assembly (Fig. 4B). These features would facilitate bacterial migration towards roots, followed by attachment and establishment^90^. Some root exudates are known to induce motility in bacteria, which is an advantageous characteristic for root colonisation, when compared to non-motile bacteria^91,92^. 16S rRNA gene-predicted functions of streptomycin, novobiocin, tetracycline and vancomycin biosynthesis were significantly enriched in the rhizosphere of semi-dwarf cultivars (Fig. 4B) and this might have some relation to the decrease in keystone taxa which resulted in a more hostile, less coordinated environment compared with tall cultivars, where microbes compete with one another in a less structured manner to gain access to rich nutrients in the rhizosphere. It should be stated that PICRUSt is a tool which provides a functional prediction of microbiome based on marker genes, but it is not an actual measurement of such functions^93^. Future work on shotgun metagenomics would enable to assess these predicted observations.

## Conclusions, Limitations and Future Directions

In recent years, there has been a growing interest in modifying plant traits to change the ability of plants to interact with beneficial microbes^13,94^. Collectively, our results showed that wheat breeding from tall to semi-dwarf plants resulted in plants less able to select and sustain a complex rhizosphere. By evaluating differences in root traits of tall and semi-dwarf wheat cultivars and the structure, composition and differential recruitment of specific taxa and keystone species, we can start to understand the impacts of breeding process on root biology. Future work should assess the effect that the breeding process has had on plant hormonal status, exudation profile, plant performance and microbiome selection under low and high nutrient supplementation. This will allow the identification of keystone species and the development of synthetic communities of increased complexity, so the significance of their presence and absence in facilitating the development of a healthy microbiome can be tested.

## Supplementary information

Supplementary information.

## Data Availability

16S rRNA gene amplicon data are available at the NCBI Sequence Read Archive (SRA) under accession number: PRJNA601112.
