# Supplementary material for: Wheat dwarfing influences selection of the rhizosphere microbiome
Source: Sci Rep. 2020 Jan 29;10:1452. doi: 10.1038/s41598-020-58402-y (PMC6989667; doi:10.1038/s41598-020-58402-y)
Supplement: Supplementary file 1 — Supplementary information. [file 41598_2020_58402_MOESM1_ESM.docx]

**SUPPLEMENTARY INFORMATION**

**Wheat dwarfing influences selection of the rhizosphere microbiome**

Vanessa N. Kavamura^1^, Rebekah J. Robinson^2^, David Hughes^3^, Ian Clark^1^, Maike Rossmann^4^, Itamar Soares de Melo^4^, Penny R. Hirsch^1^, Rodrigo Mendes^4^, Tim H. Mauchline^1*^

^1^Sustainable Agriculture Sciences, Rothamsted Research, Harpenden, Hertfordshire, United Kingdom.

^2^Plant Pathology Laboratory, Royal Horticultural Society, RHS Garden Wisley, Woking, Surrey, GU23 6QB, United Kingdom.

^3^Computational and Analytical Sciences, Rothamsted Research, Harpenden, Hertfordshire, United Kingdom.

^4^ Laboratory of Environmental Microbiology, Embrapa Environment, Jaguariúna-SP, Brazil.

^*^tim.mauchline@rothamsted.ac.uk

**SUPPLEMENTARY METHODS**

- Details of root morphological traits analysis
- Details of Illumina bacterial 16S rRNA gene sequencing
- Details of 16S rRNA gene amplicon sequence analysis
- Details of Microbiome Analyst analysis
- Details of network analysis
- Details of PICRUSt analysis

**SUPPLEMENTARY FIGURES**

Supplementary Figure S1. Average height (cm) of tall and semi-dwarf wheat plants (n = 7) grown in pots under glasshouse conditions.

Supplementary Figure S2. Root morphological traits obtained for tall and semi dwarf cultivars.

Supplementary Figure S3. Relative abundance (%) (>1%) of 16S rRNA gene derived bacterial sequences of rhizosphere from wheat cultivars classified at phylum level.

Supplementary Figure S4. Alpha diversity metrics of 16S rRNA gene data of wheat rhizosphere obtained tall and semi-dwarf cultivars.

Supplementary Figure S5. Modular organization of phylogenetic molecular ecological networks (pMENs) obtained for tall (A) and semi-dwarf (B) cultivars.

Supplementary Figure S6. Bacterial composition at phylum level of modules with more than 10 nodes.

**SUPPLEMENTARY TABLES**

Supplementary Table S1. Properties obtained after network analysis.

**SUPPLEMENTARY METHODS**

***Details of root morphological traits analysis***

Four uniformly-germinated seeds were placed on one germination paper (25 × 38 cm, Anchor Paper Company, St. Paul, MN, USA), which was placed in the upright position in a tray with nutrient solution (1.5 mM Ca(NO_3_)_2_, 5 mM KNO_3_, 2 mM NaNO_3_, 1 mM MgSO_4_, 1 mM KH_2_PO_4_, 25 μM FeEDTA, 160 nM CuCl_2_, 9.2 μM H_3_BO_3_, 3.6 μM MnCl_2_, 16 nM Na_2_MoO_4_, 5 μM KCl, and 770 nM ZnCl_2_, at pH 5.8). A total of three paper rolls were obtained for each cultivar. Initially the solution was half-strength and after three days, it was changed to full-strength medium and thereafter changed every day with full-strength medium. Plants were kept in a growth chamber (Sanyo MLR 350) (12/12 h light/dark cycle, light intensity of 500 μmol.m^–2^.s^–1^, temperature of 20°C during the day and 16°C during darkness). After eleven days, seedlings were removed from the germination paper and preserved in 30% ethanol prior to imaging. The intact root systems were scanned as a digital image with a scanner (STD-1600, Regent Instruments, QC, Canada). The total root length, surface area, volume, diameter and density were determined from the root images using the root image analysis software WinRHIZO Pro (Regent Instruments).

***Details of Illumina bacterial 16S rRNA gene sequencing***

The V4 region of the 16S rRNA gene (515F-806R) was PCR-amplified with region-specific primers that include sequencer adapter sequences used in the Illumina flowcell [32, 95]. The reverse amplification primer contained a twelve-base barcode sequence that supports pooling of up to 2,167 different samples in each lane [32, 95]. Each 25 µL PCR reaction contained 12 µL of MO BIO PCR Water (Certified DNA-Free), 10 µL of 5 Prime HotMasterMix (2.5x concentration, 1x final), 1 µL Forward Primer (5 µM concentration, 200 pM final), 1 µL Reverse Primer (5 µM concentration, 200 pM final), and 1 µL of template DNA. The conditions for PCR were as follows: 94°C for 3 minutes to denature the DNA, with 35 cycles at 94°C for 45 s, 50°C for 60 s, and 72°C for 90 s; with a final extension of 10 min at 72°C to ensure complete amplification. Amplicons were then quantified using PicoGreen (Invitrogen) and a plate reader (Infinite® 200 PRO, Tecan). Once quantified, volumes of each of the products were pooled into a single tube so that each amplicon was represented in equimolar amounts. This pool was cleaned up using the UltraClean® PCR Clean-Up Kit (MO BIO), and then quantified using a fluorometer (Qubit, Invitrogen). After quantification, the molarity of the pool was determined and diluted down to 2 nM, denatured, and then diluted to a final concentration of 6.75 pM with a 10% PhiX spike for sequencing on the Illumina MiSeq. Amplicons were sequenced on a 151bp x 12bp x 151bp MiSeq run using customized sequencing primers and procedures [32].

***Details of 16S rRNA gene amplicon sequence analysis***

**Instruction:** Create these three files (“**config_file**”, “**steps_commands**” and “**16S_pipeline.sh**”) in the folder where you want to perform your analysis.

**File: “config_file”**

# $PWD = represents the location of your files and downloaded scripts

FORWARD_READS=$PWD/Undetermined_S0_L001_R1_001.fastq

REVERSE_READS=$PWD/Undetermined_S0_L001_R2_001.fastq

BARCODES_FASTQ=$PWD/Undetermined_S0_L001_I1_001.fastq

MAPPING=$PWD/mapping_file.txt

#

BIOM=$PWD/Scripts/biom-format-2.0.1/scripts/biom

#available at: <http://biom-format.org/index.html>

BMP_UPARSE=$PWD/Scripts/bmp-Qiime2Uparse.pl

#available at: <https://github.com/vpylro/BMP>

E_VALUE= x

# x = E_VALUE to be determined

BMP_RENAME=$PWD/Scripts/bmp-otuName.pl

#available at: <https://github.com/vpylro/BMP>

BMP_CONVERT=$PWD/Scripts/bmp-map2qiime.py

#available at: <https://github.com/vpylro/BMP>

#

SILVA128_REP_SET=$PWD/Silva128/rep_set/rep_set_16S_only/97/97_otus_16S.fasta

SILVA128_TAXONOMY=$PWD/Silva128/taxonomy/16S_only/97/consensus_taxonomy_7_levels.txt

SILVA128_ALIGNMENT=$PWD/Silva128/core_alignment/core_alignment_SILVA128.fna

#SILVA128 available at: <https://www.arb-silva.de/documentation/release-128/>

ID=97

RUN=x

# (you can choose any name for your analysis)

USEARCH9=$PWD/Scripts/usearch9.2.64_i86linux32

#USEARCH9.2.64 – You might need 64-bit version for large files

#available at: <https://www.drive5.com/usearch/>

FASTA_FORMATTER=$PWD/fastx_toolkit-0.0.13.2/src/fasta_formatter/fasta_formatter

#available at: <http://hannonlab.cshl.edu/fastx_toolkit/>

ALPHA_PARAMS_FILE=/home/data/mmeg_ngs/Scripts/alpha_params.txt

#file in .txt format with the alpha diversity indexes you want to use. E.g.: alpha_diversity:metrics shannon,PD_whole_tree,chao1,observed_species,fisher_alpha

###

**File: “steps_commands”**

STEP=1

COMMAND="validate_mapping_file.py \

-m ${MAPPING} \

-o ${RUN}/validate_mapping_output \

"

run_command

STEP=2

COMMAND="join_paired_ends.py \

-f ${FORWARD_READS} \

-r ${REVERSE_READS} \

-b ${BARCODES_FASTQ} \

-o ${RUN}/joined_output \

"

run_command

STEP=3

COMMAND="split_libraries_fastq.py \

-i ${RUN}/joined_output/fastqjoin.join.fastq \

-b ${RUN}/joined_output/fastqjoin.join_barcodes.fastq \

-m ${MAPPING} \

--store_demultiplexed_fastq \

--rev_comp_mapping_barcodes \

-q 0 -n 100 -r 0 --barcode_type 12 \

-o ${RUN}/split_libraries_output \

"

run_command

STEP=4

COMMAND="if [[ ! -e ${RUN}/fasta ]]; then

mkdir -v ${RUN}/fasta; else

echo Directory ${RUN}/fasta exists; fi;

if [[ ! -e ${RUN}/uclust_mapping ]]; then

mkdir -v ${RUN}/uclust_mapping; else

echo Directory ${RUN}/uclust_mapping exists; fi

"

run_command

STEP=5

COMMAND="${USEARCH9} \

-fastq_filter ${RUN}/split_libraries_output/seqs.fastq \

-fastq_maxee 1.0 \

-fastq_trunclen 240 \

-fastaout ${RUN}/fasta/reads.fa \

"

run_command

STEP=6

COMMAND="perl ${BMP_UPARSE} \

-i ${RUN}/fasta/reads.fa \

-o ${RUN}/fasta/reads_bmp.fa \

"

run_command

STEP=7

COMMAND="${USEARCH9} \

-fastx_uniques ${RUN}/fasta/reads_bmp.fa \

-fastaout ${RUN}/fasta/unique.fa \

-sizeout \

-threads 16 \

"

run_command

STEP=8

COMMAND="${USEARCH9} \

-sortbysize ${RUN}/fasta/unique.fa \

-fastaout ${RUN}/fasta/sorted.fa \

-minsize 2 \

"

run_command

STEP=9

COMMAND="${USEARCH9} \

-cluster_otus ${RUN}/fasta/sorted.fa \

-otus ${RUN}/fasta/otus_unformatted.fa \

"

run_command

STEP=10

COMMAND="${FASTA_FORMATTER} \

-i ${RUN}/fasta/otus_unformatted.fa \

-o ${RUN}/fasta/otus_formatted.fa \

"

run_command

STEP=11

COMMAND="perl ${BMP_RENAME} \

-i ${RUN}/fasta/otus_formatted.fa \

-o ${RUN}/fasta/otus.fa \

"

run_command

STEP=12

COMMAND="${USEARCH9} \

-usearch_global ${RUN}/fasta/reads_bmp.fa \

-db ${RUN}/fasta/otus.fa \

-strand both \

-id 0.${ID} \

-uc ${RUN}/uclust_mapping/map.uc \

-threads 16 \

"

run_command

STEP=13

COMMAND="python ${BMP_CONVERT} ${RUN}/uclust_mapping/map.uc"

run_command ${RUN}/otu_table.txt

STEP=14

COMMAND="assign_taxonomy.py \

-i ${RUN}/fasta/otus.fa \

-o ${RUN}/assigned_taxonomy_output \

-r $SILVA128_REP_SET \

-t $SILVA128_TAXONOMY \

"

run_command

STEP=15

COMMAND="align_seqs.py \

-i ${RUN}/fasta/otus.fa \

-o ${RUN}/rep_set_aligned \

-t $SILVA128_ALIGNMENT \

"

run_command

STEP=16

COMMAND="filter_alignment.py \

-i ${RUN}/rep_set_aligned/otus_aligned.fasta \

-o ${RUN}/filtered_alignment \

"

run_command

STEP=17

COMMAND="make_phylogeny.py \

-i ${RUN}/filtered_alignment/otus_aligned_pfiltered.fasta \

-o ${RUN}/filtered_alignment/otus_aligned_pfiltered_rep_set.tre \

"

run_command

STEP=18

COMMAND="make_otu_table.py \

-i ${RUN}/otu_table.txt \

-t ${RUN}/assigned_taxonomy_output/otus_tax_assignments.txt \

-o ${RUN}/otu_table_tax.biom \

"

run_command

STEP=19

COMMAND="${BIOM} \

summarize-table \

-i ${RUN}/otu_table_tax.biom \

-o ${RUN}/biom_summary \

"

run_command

STEP=20

COMMAND="core_diversity_analyses.py \

-i ${RUN}/otu_table_tax.biom \

-m ${MAPPING} \

-t ${RUN}/filtered_alignment/otus_aligned_pfiltered_rep_set.tre \

-e ${E_VALUE} \

-o ${RUN}/diversity_${E_VALUE} \

-p ${ALPHA_PARAMS_FILE}

"

run_command

STEP=21

COMMAND="${BIOM} \

convert \

-i ${RUN}/otu_table_tax.biom \

-o ${RUN}/otu_table_tax_converted_from_biom.txt \

-b \

--header-key="taxonomy" \

--table-type="OTU_table" \

"

run_command

###

**File: “16S_pipeline.sh”**

#!/bin/bash

#

# Script to run selected steps from a pipeline

# ############################################

# Last modified April 2017

#

# Used here to run a multi-stage QIIME analysis.

#

# Just now, you need to run one of the following commands first.

# activate-qiime-1.8.0.sh

# activate-qiime-1.9.0.sh

# They set up a suitable environment for your qiime commands.

#

# The arguments to this script are the steps to be run.

# Parameters and filenames are in file: config_file

# Commands for each step are in file: steps_commands

#

# Modify config_file with the correct location of “FORWARD_READS”, “REVERSE_READS”, BARCODES_FASTQ”, “MAPPING”. Change the “RUN” name as desired.

#

# Suggested commands:

# activate-qiime-1.8.0.sh (step 1 doesn’t work with qiime 1.9.0)

# bash 16S_pipeline.sh 1

# activate-qiime-1.9.0.sh

# bash 16S_pipeline.sh 2 3 4 5 6 7 8 9 10 11 12 13 14 15 16 17 18 19

# Open the biom_summary file and check the min value

# Change “E_VALUE” in config_file to this value

# activate-qiime-1.8.0.sh (step 20 doesn’t work with qiime 1.9.0)

# bash 16S_pipeline.sh 20 21

#

#

##############################

# Function to parse arguments

##############################

# Example arguments: 1 4 5-8

# Returns: 1 4 5 6 7 8

# Code taken from the 'qjobs' awk script

#

function get_steps {

PARSED=$(echo $* | awk ' {

PotentialNumbers = ""

for (num = 1; num <= NF; num++) {

PotentialNumber = $num

if (PotentialNumber ~ /^[0-9]+$/) {

PotentialNumbers = sprintf("%s %s", PotentialNumbers, PotentialNumber)

} else {

gsub("[^-0-9]", "", PotentialNumber)

}

if (PotentialNumber ~ /^[0-9]*-+[0-9]*$/) {

split(PotentialNumber, ARRAY, "-+")

StartNumber = ARRAY[1] + 0

EndNumber = ARRAY[2] + 0

if (EndNumber == 0) { EndNumber = StartNumber }

if (StartNumber == 0) { StartNumber = 1 }

Command = "seq " StartNumber " " EndNumber

while(Command | getline PotentialNumber) {

PotentialNumbers = sprintf("%s %s", PotentialNumbers, PotentialNumber)

}

close(Command)

}

}

Command = "echo " PotentialNumbers " | tr \" \" \"\n\" | sort -un | tr \"\n\" \" \""

Command | getline PotentialNumbers

close(Command)

# print "PotentialNumbers = \"" PotentialNumbers "\""

print PotentialNumbers

}')

# return value to std out

echo "$PARSED"

}

##########################################

# Function to run command for given STEP

##########################################

#

function run_command {

if ( echo $STEPS | fgrep -wq ${STEP})

then

printf " Running step %2d:\n\t${COMMAND}\n\n" ${STEP}

if [[ -z ${1} ]]

then

#${1} is null or unset

eval ${COMMAND}

else

#redirect std output to ${1}

${COMMAND} > ${1}

fi

# Messy, but added to allow standard output redirection.

# Can probably be collapsed into a single if-then-else using eval.

else

# printf "Skipping step %2d:\n\t${COMMAND}\n\n" ${STEP}

printf "Skipping step %2d:\n" ${STEP}

fi

# status of the last executed command is $?

check_stage $?

}

############################

# Other Function Definitions

############################

#

# quit if any step fails

function check_stage {

if [[ ${1} -ne 0 ]]

then

echo "ERROR in step ${STEP}"

exit_script ${STEP}

else

STEP=$((STEP+1))

fi

}

#

# exit gracefully

function exit_script {

# forcibly delete any TMPDIR (if you own them)

if [[ ( -n ${TMPDIR} ) && ( -d ${TMPDIR}) && ( -O ${TMPDIR} ) ]]

then

rm -rvf ${TMPDIR}

fi

exit

}

#

# Make a temporary directory

function make_tmpdir {

export TMPDIR=$(mktemp -d)

# is it writable?

if [[ ! -w ${TMPDIR} ]]

then

printf "\n"

echo -e "Unable to create temporary directory!\nPlease Investigate.\n"

exit_script

fi

}

#############################

# End of Function Definitions

#############################

#############################

# Start Preliminaries

#############################

# Before we start, we are at STEP 0

STEP=0

# call exit_script on interrupt

trap exit_script INT KILL STOP

# Some scripts from this directory are needed?

export PATH=${PATH}:/home/data/mmeg_ngs/Scripts

# Make TMPDIR (if needed)

##make_tmpdir

# get steps to run

STEPS="$(get_steps $*)"

echo -e "Steps selected: "$STEPS"\n"

#############################

# Read config_file

#############################

source ./config_file

echo "The config_file is"

cat ./config_file

echo "###End config_file"

echo -e "Output prefix is:"$RUN"\n"

# Could add tests that files exist, checks on other settings, etc

source ./steps_commands

exit_script

###

***Details of Microbiome Analyst analysis***

The filtered OTU was arranged as the required format and it was uploaded with the mapping and taxonomy files. Low abundance and low variance OTUs were removed using default values, where OTUs with less than 2 counts in less than 20% of the samples and 10% of the values below the determined inter-quantile range (IQR) were removed. The OTU table was normalised using the method of rarefying and relative log-expression (RLE) transformed.

***Details of network analysis***

Firstly, the OTU table was filtered using the command *filter_otus_from_otu_table.py* (--min_count_fraction 0.0001) in QIIME to retain all OTUs with at least 0.01% of total abundance. Since the numbers of sequences of all samples were diverse, OTU counts were transformed into relative abundance by dividing the sum of each sample [96]. The relative abundance table was split into two datasets: one dataset for tall cultivars and another dataset for semi-dwarf cultivars. Each dataset was manually filtered to exclude OTUs which did not occur in at least 65% of all samples, OTUs which satisfied this criterion were kept for upload into MENA pipeline. Network construction was performed using default values except for two settings: majority was set to 1 and logarithm was not taken. Random Matrix Theory (RMT) was used to automatically identify the appropriate threshold value prior to network construction. Modules were detected by fast greedy modularity optimization [97] and this helped identify OTUs (nodes) that potentially play structural roles in rhizosphere networks associated to tall and semi-dwarf cultivars. The concepts of within-module connectivity (Zi) and between-module connectivity (Pi) [75] were used and nodes were separated into four categories: module hubs, network hubs, connectors and peripherals [41, 69]. The ratio of positive (P) to negative (N) co-occurrence patterns (P: N ratio) was calculated [98] to provide information about global behaviour of taxa [99].

***Details of PICRUSt analysis***

PICRUSt relies on the topology of the tree and the distance to the next sequenced organism, using the nearest neighbour identification based on a minimum 16S rRNA gene sequence similarity. An OTU table was created with a closed-reference picking protocol using the GreenGenes database (version 13_8) at 97% identity in QIIME. Next, the OTU table was uploaded into GALAXY and it was normalised to take into account 16S rRNA gene copy numbers to reflect OTU abundances. The ‘predict metagenome’ module was then used to create a square matrix with normalised OTU abundance and multiplied by functional trait abundance per sample. The nearest sequenced taxon index (NSTI) was calculated to estimate whether reference genome coverage was sufficient for accurate metagenome assignment, with small values representing high accuracy [44]. ‘Categorize by function’ module was used to create a predicted metagenome functional content table with Kyoto Encyclopaedia of Genes and Genomes (KEGG) to a level 3 hierarchy.

**SUPPLEMENTARY FIGURES**


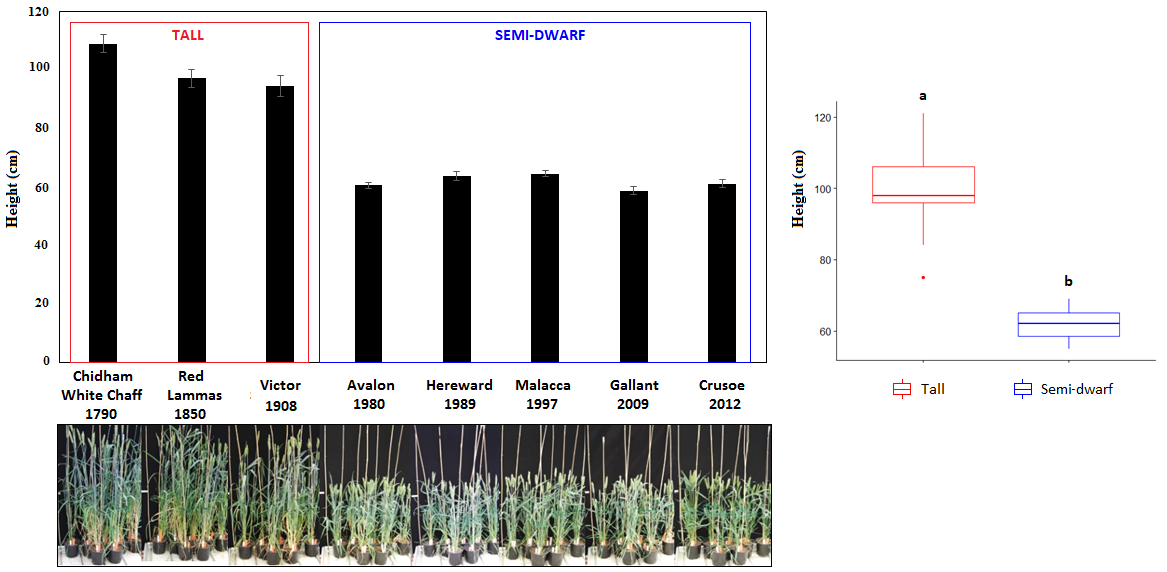


**Supplementary Figure S1**. **Average height (cm) of tall and semi-dwarf wheat plants (n = 7) grown in pots under glasshouse conditions.** Tall cultivars are represented by Chidham White Chaff, Red Lammas and Victor, whereas semi-dwarf cultivars include Avalon, Hereward, Malacca, Gallant and Crusoe. There were statistically significant differences between the height means of tall and semi-dwarf cultivars as determined by ANOVA (p < 0.05).


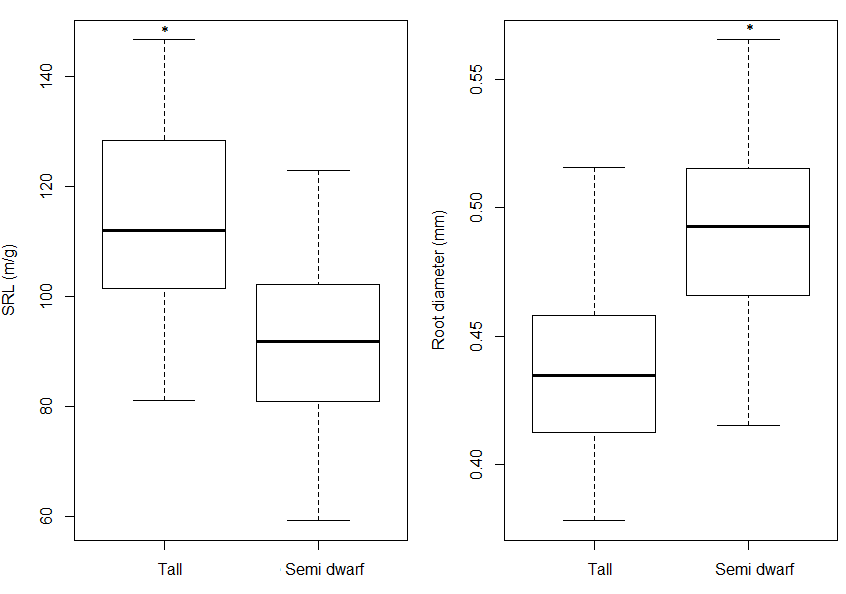


**Supplementary Figure S2. Root morphological traits obtained for tall and semi dwarf cultivars.** **(a)** Specific root length (SRL) which is the product of root length divided by root dry weight and **(b)** Root diameter. Asterisks show statistically significant differences calculated using ANOVA (p < 0.05).


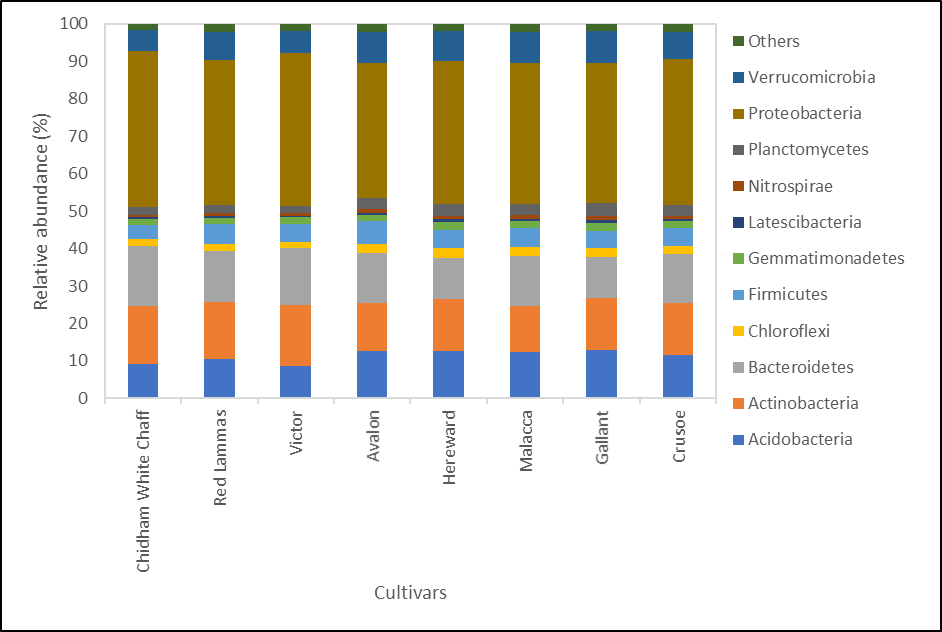


**Supplementary Figure S3.** **Relative abundance (%) (>1%) of 16S rRNA gene derived bacterial sequences of rhizosphere from wheat cultivars classified at phylum level.** Others include thirty-one phyla with relative abundance lower than 1%: Armatimonadetes, BRC1, Candidatus Berkelbacteria, Chlamydiae, Chlorobi, Cloacimonetes, Cyanobacteria, Deferribacteres, Deinococcus-Thermus, Elusimicrobia, FBP, FCPU426, Fibrobacteres, GAL15, Gracilibacteria, Hydrogenedentes, Ignavibacteriae, Marinimicrobia (SAR406 clade), Microgenomates, Omnitrophica, PAUC34f, Parcubacteria, Peregrinibacteria, RBG-1, (Zixibacteria), Saccharibacteria, Spirochaetae, TM6 (Dependentiae), Tectomicrobia, Tenericutes, WS2 and WWE3.


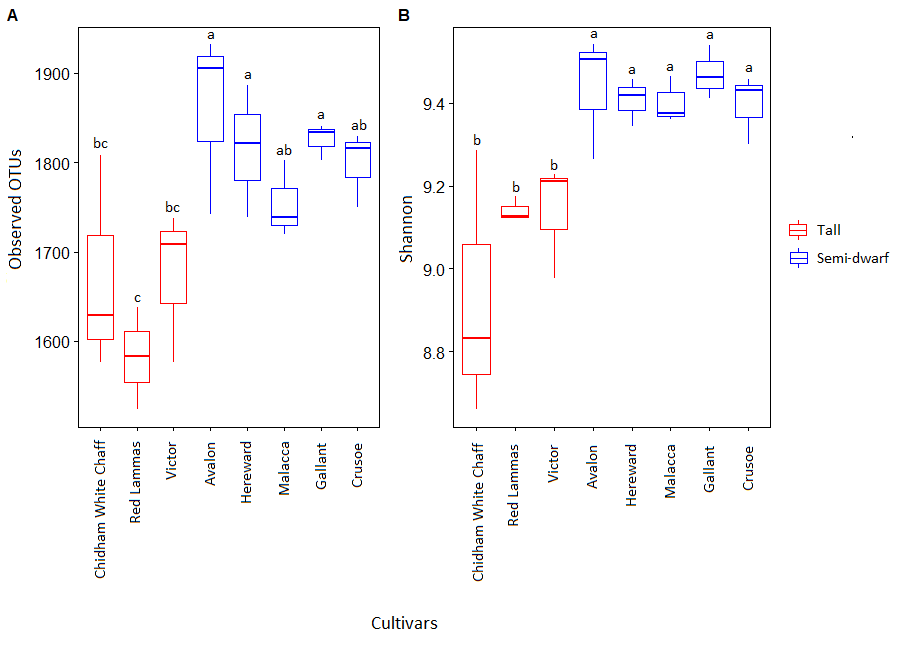


**Supplementary Figure S4.** **Alpha diversity metrics of 16S rRNA gene data of wheat rhizosphere obtained tall and semi-dwarf cultivars.** Cultivars are grouped per height, with red colour representing tall cultivars and blue colour representing semi-dwarf cultivars. (**A**) Observed OTUs and (**B**) Shannon. Different letters denote statistically significant differences according to Fisher’s LSD test (p < 0.05).


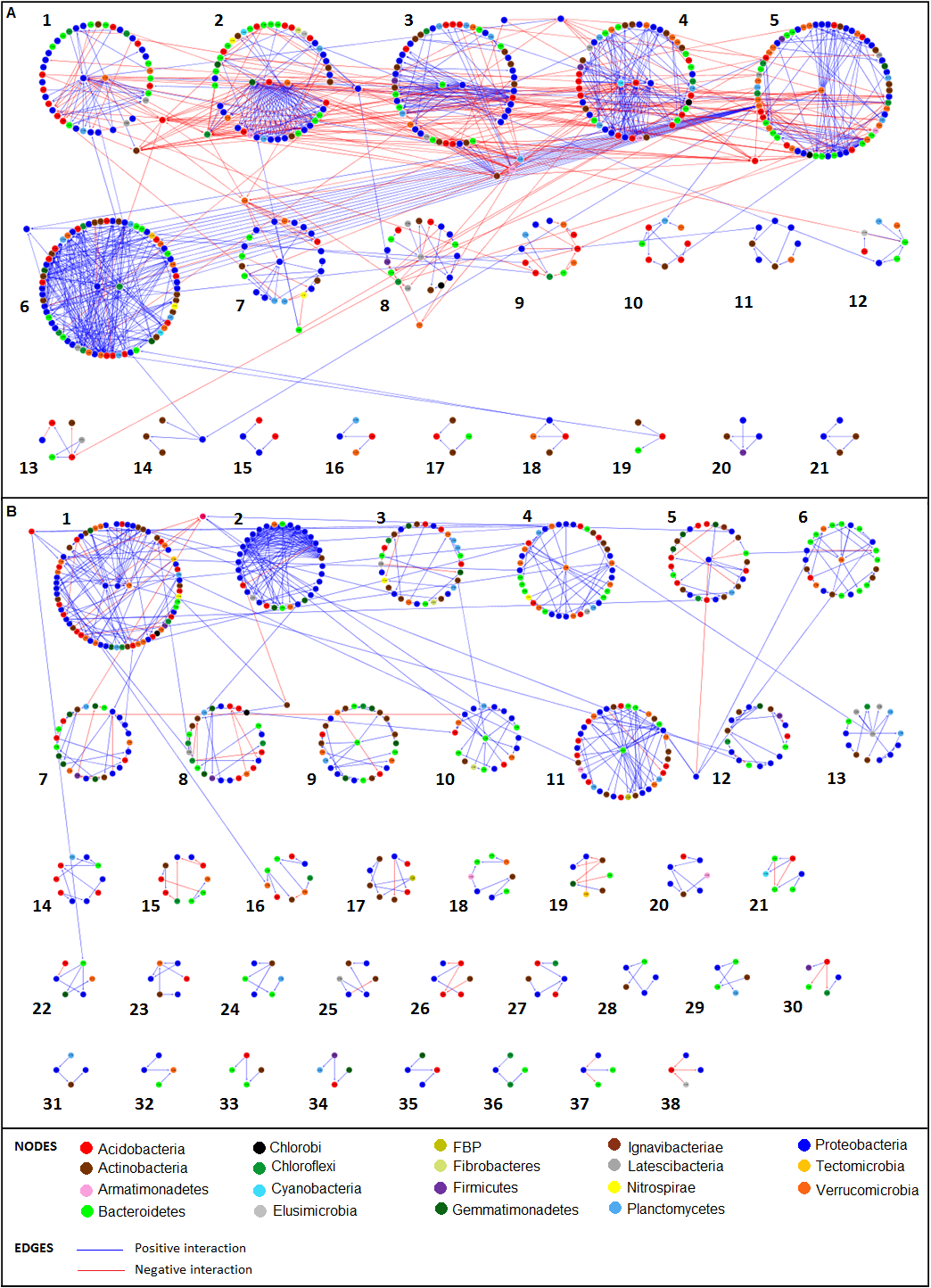


**Supplementary Figure S5.** **Modular organization of phylogenetic molecular ecological networks (pMENs) obtained for tall (A) and semi-dwarf (B) cultivars.** Only modules with 4 or more nodes are shown. Each node (dot) represents an OTU and each colour indicates one respective phylum. The edges are represented by lines, with positive co-occurrence patterns between two nodes shown in blue and negative co-occurrence patterns shown in red. Nodes inside the modules represent module hubs and nodes outside and a little bit distant from their modules represent the connectors, both considered keystone OTUs.


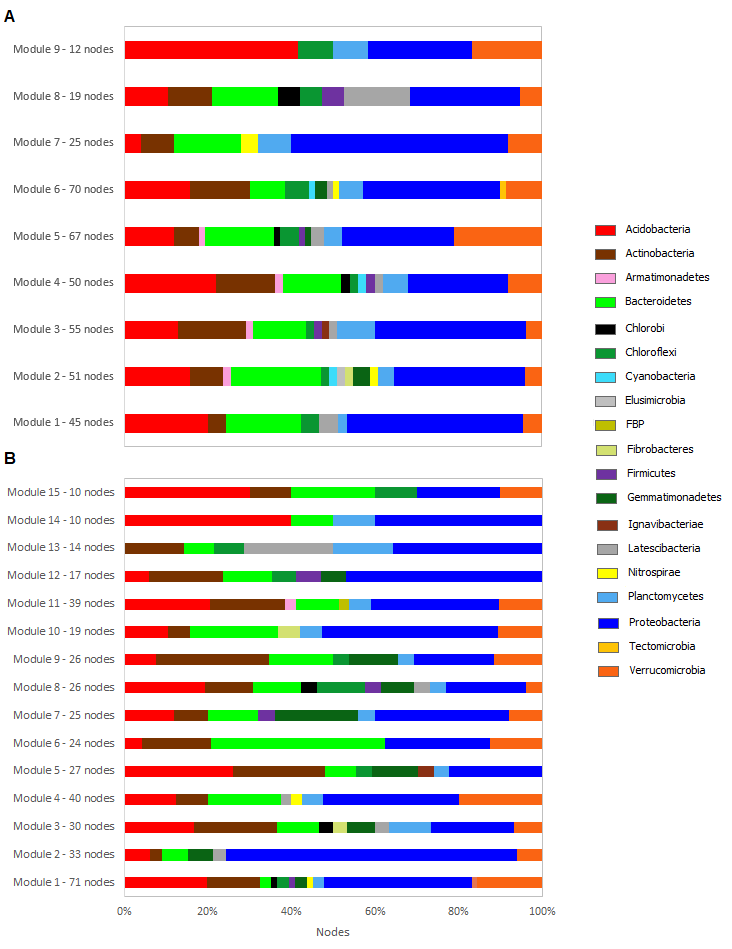


**Supplementary Figure S6.** **Bacterial composition at phylum level of modules with more than 10 nodes.** The bars show the percentage of the total number of nodes for each module for tall (**A**) and semi-dwarf cultivars (**B**).

**SUPPLEMENTARY TABLES**

**Supplementary Table S1.** **Properties obtained after network analysis.** Major topological properties (*) of phylogenetic molecular ecological networks (pMENs) of bacterial communities of wheat rhizosphere from tall and semi-dwarf cultivars.

| **Cultivars** | **Tall** | **Semi-dwarf** |
| --- | --- | --- |
| Number of OTUs^a^ | 1233 | 1147 |
| Number of nodes | 615 | 705 |
| Number of links | 1175 | 842 |
| R^2^ of power-law^b^ | 0.907 | 0.899 |
| Average path distance (GD)^c^ | 5.026 | 9.187 |
| Average degree (avgK)^d^ | 3.821 | 2.389 |
| Modularity^e^ | 0.749 | 0.890 |
| Number of modules | 94 | 115 |
| Number of positive interactions (P) | 888 | 709 |
| Number of negative interactions (N) | 287 | 133 |
| Ratio (P:N) | 3.094 | 5.331 |

^a^ Number of OTUs originally used for network construction with RMT-based approach

^b^ Indicates a scale-free characteristic used to describe the finding that most nodes in a network have few neighbours while few nodes have large number of neighbours. In most cases, the connectivity distribution asymptotically follows a power law model

^c^ Geodesic distance. A smaller GD means all nodes in the network are closer

^d^ High avgK means a more complex network

^e^ Modularity values range from 0 to 1 and are used to measure how well a network is able to be separated into modules

*For more details about each property, please refer to Deng et al. [41].

**ADDITIONAL REFERENCES**

1. Caporaso, J.G. *et al.* Global patterns of 16S rRNA diversity at a depth of millions of sequences per sample. *PNAS.* 108(1), 4516-4522 (2011).
2. Zhou, J. *et al.* Reproducibility and quantitation of amplicon sequencing-based detection. *ISME J.* **5**, 1303-1313 (2011).
3. Newman, M.E.J. Fast algorithm for detecting community structure in networks. *Phys. Rev.* **69**, 066133-1-066133-5 (2004).
4. Xun, W. *et al.* Alteration of soil bacterial interaction networks driven by different long-term fertilization management practices in the red soil of South China. *Appl. Soil Ecol.* **120**, 128-134 (2017).
5. Karimi, B. *et al.* Microbial diversity and ecological networks as indicators of environmental quality. *Environ. Chem. Lett.* **15**, 265-281 (2017).
